# The Role of MicroRNAs in the Pathophysiology and Management of Heart Failure: From Molecular Mechanisms to Clinical Application

**DOI:** 10.3390/ijms262412085

**Published:** 2025-12-16

**Authors:** Irina Gilyazova, Yanina Timasheva, Anna Chumakova, Gulshat Abdeeva, Marina Plotnikova, Naufal Zagidullin

**Affiliations:** 1 Department of Medical Genetics and Fundamental Medicine, Faculty of General Medicine, Bashkir State Medical University, 450008 Ufa, Russia; 2 Institute of Biochemistry and Genetics, Ufa Federal Research Centre of Russian Academy of Sciences, 450054 Ufa, Russia; 3 Department of Pathological Physiology, Faculty of General Medicine, Bashkir State Medical University, 450008 Ufa, Russia; gulshatik2001@mail.ru; 4 Department of Cardiology, Clinical Hospital of the Bashkir State Medical University, 450008 Ufa, Russia; plotnikovam.r@yandex.ru; 5 Department of Propaedeutics of Internal Diseases, Faculty of General Medicine, Bashkir State Medical University, 450008 Ufa, Russia; znaufal@mail.ru

**Keywords:** heart failure, heart failure with preserved ejection fraction, non-coding RNAs, microRNAs

## Abstract

Heart failure (HF) remains a leading cause of morbidity and mortality worldwide, affecting over 30 million individuals, with its prevalence steadily increasing due to population aging. Among its forms, heart failure with preserved ejection fraction (HFpEF) has emerged as a major clinical and public health concern, now accounting for more than half of all HF cases and closely associated with comorbidities such as hypertension, obesity, and diabetes. MicroRNAs (miRNAs) have gained recognition as key regulators of the molecular mechanisms underlying HF, particularly HFpEF, where they modulate interconnected pathways of inflammation, fibrosis, and endothelial dysfunction. This review discusses the mechanisms by which miRNAs contribute to the pathogenesis of HF and examines their potential as both biomarkers and therapeutic targets. By integrating current evidence, it aims to clarify the prognostic significance and clinical applicability of these molecular markers, highlighting their role in advancing personalized strategies for the diagnosis and management of HF.

## 1. Introduction

**Figure 1:**
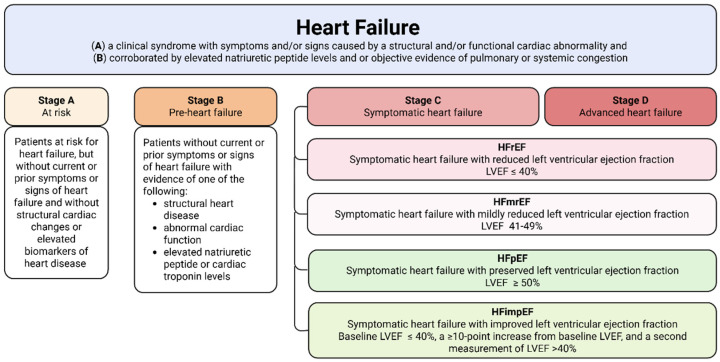
Classification of heart failure. The contemporary classification of heart failure (HF) is based on the universal definition, which identifies HF as a clinical syndrome arising from structural or functional cardiac abnormalities accompanied by symptoms, signs, elevated natriuretic peptides, or objective evidence of congestion. The staging framework reflects symptom onset and progression, distinguishing individuals at risk (Stage A), with pre-HF (Stage B), with symptomatic HF (Stage C), and with advanced disease (Stage D). Functional status is evaluated using the NYHA classification, and HF is further categorized by left ventricular ejection fraction into HFrEF (≤40%), HFmrEF (41–49%), HFpEF (≥50%), and HFimpEF, defined by an improvement from ≤40% to a value above 40%.

Heart failure (HF) remains a major global cause of mortality and hospitalization, with prevalence rising as populations age. More than 30 million individuals are currently affected, and the number continues to increase [1]. Despite ongoing efforts, a uniform definition of HF in clinical and research practice is not always straightforward. The universal definition describes HF as a clinical syndrome characterized by symptoms or signs resulting from structural or functional cardiac abnormalities, supported by elevated natriuretic peptides (NT-proBNP) or objective evidence of congestion. Current staging emphasizes symptom onset and progression (Figure 1), distinguishing individuals at risk (Stage A), with pre-HF (Stage B), with symptomatic HF (Stage C), and with advanced disease (Stage D). Functional capacity is commonly assessed through the New York Heart Association (NYHA) functional classification, which grades patients from Class I (no limitation of physical activity) to Class IV (symptoms at rest or minimal activity). HF is further categorized by left ventricular ejection fraction (LVEF) into reduced (HFrEF, ≤40%), mildly reduced (HFmrEF, 41–49%), preserved (HFpEF, ≥50%), and improved (HFimpEF), the latter defined by a transition from an LVEF ≤ 40%, to a value above >40% following a substantial increase [2,3].

HFpEF arises through diverse mechanisms, most frequently involving left ventricular injury due to coronary artery disease (CAD), myocarditis, or valvular pathology. It represents a heterogenous clinical syndrome characterized by a high burden of comorbidities and multisystem involvement, including vascular dysfunction, skeletal muscle abnormalities, and alterations in body composition. Although the biological diversity of HF is increasingly recognized, the fundamental processes distinguishing HFpEF from HFrEF remain incompletely understood. Transitions between LVEF categories are common, and patients with HFmrEF who progress to HFrEF carry a poorer prognosis than those who remain stable or move toward a higher LVEF category [4]. Those transitioning from HFpEF to HFrEF generally exhibit more complex clinical profiles and more severe diastolic dysfunction. Evidence also suggests that HFmrEF may represent a mixed phenotype that can be further refined on the basis of LVEF stability [5]. Meta-analytic data consistently show lower adjusted mortality in HFpEF than in HFrEF [6].

The marked clinical diversity of HF reflects the interplay of numerous risk factors that activate distinct pathophysiological mechanisms to varying degrees. Microvascular inflammation, endothelial dysfunction, fibrosis, mitochondrial impairment, and autonomic imbalance contribute to functional and structural remodeling at multiple biological levels [7,8]. These processes underscore the need for more effective preventive and therapeutic strategies as HF continues to impose a growing global burden.

Genetic factors play an important role in HF susceptibility and progression. Both rare, highly penetrant variants and numerous common variants exert measurable effects. A recent exome-wide association study involving more than 8000 participants across clinical trials and population cohort identified several genes (*FAM221A*, *CUTC*, *IFIT5*, *STIMATE*, *TAS2R20*, *CALB2*, *BLK*) in which rare pathogenic variants were associated with adverse HF outcomes [9,10]. Large genome-wide association studies (GWAS) have further confirmed the polygenic architecture of HF and related cardiometabolic traits [11,12]. In a meta-analysis of more than 47,000 HF cases, twelve independent variants across eleven genomic loci were linked to HF, many of them shared with CAD, atrial fibrillation (AF), or impaired ventricular function. Genes involved in cardiac development (*MYOZ1*, *SYNPO2L*), protein homeostasis (*BAG3*), and cellular senescence (*CDKN1A*) appear to be particularly relevant. These insights highlight the value of genetic information for refining diagnosis and improving patient management, especially in inherited cardiomyopathies [13].

Polygenic risk scores (PRSs) capture the cumulative effect of multiple HF-associated variants and have demonstrated predictive value for several cardiovascular conditions [14,15]. Scores derived from 238 genetic variants influencing ventricular geometry or associated with chronic HF improved risk estimation beyond traditional clinical predictors [16]. Integrating PRS with established factors, including NT-proBNP levels, enhances prognostic accuracy [17], suggesting a potential role for such tools in future risk-stratification models [18,19].

**Figure 2:**
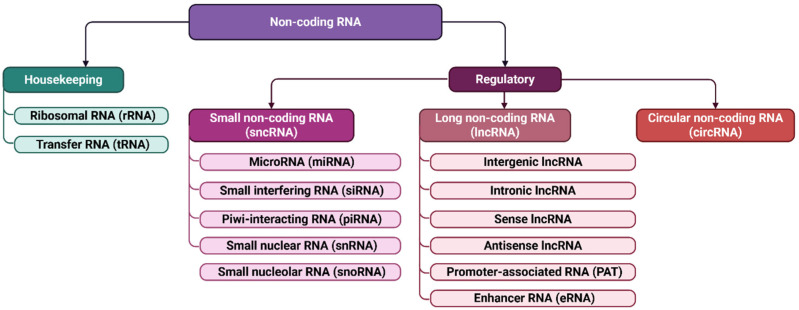
Non-coding RNAs classification. Non-coding RNAs are classified according to their function and size. This includes housekeeping RNAs, such as ribosomal (rRNA) and transfer (tRNA) RNAs, as well as regulatory non-coding RNAs. Regulatory RNAs are further subdivided into small non-coding RNAs, up to 200 nucleotides in length—including microRNAs (miRNAs), small nuclear RNAs (snRNAs), small nucleolar RNAs (snoRNAs), small interfering RNAs (siRNAs), and PIWI-interacting RNAs (piRNAs)—and long non-coding RNAs (lncRNAs), which exceed 200 nucleotides.

Growing evidence underscores the pivotal role of non-coding RNAs in HF pathogenesis. Regulatory non-coding RNAs, including microRNAs (miRNAs), small interfering RNAs (siRNAs), and long non-coding RNAs (lncRNAs) (Figure 2), participate in chromatin regulation, transcriptional control, and post-transcriptional gene silencing. MiRNAs are particularly relevant, as they modulate apoptosis, inflammatory signaling, fibrosis, angiogenesis, and metabolic remodeling [20,21,22,23]. Their involvement begins early in cardiac development, where dysregulation can lead to congenital defects [24,25], and continues throughout life in processes governing myocardial injury and repair [24,26,27]. Mature cardiomyocytes lose their proliferative capacity after birth, so perturbations in miRNA networks can contribute to irreversible damage and progressive ventricular dysfunction.

MiRNAs also influence individual susceptibility to hypertrophy, fibrosis, and other maladaptive processes that culminate in HF [28,29]. Aberrant expression patterns correlate with disease progression, offering opportunities for both diagnostic and therapeutic innovation. Ongoing work aimed at characterizing the miRNA transcriptome, elucidating regulatory pathways, and identifying HF-specific miRNAs is essential for developing personalized approaches to monitoring and treating HF [28].

This review focuses on the mechanisms through which miRNAs contribute to HF pathogenesis and examines their potential as biomarkers and therapeutic targets.

## 2. Materials and Methods

This review was structured to synthesize current understanding of the mechanisms by which miRNAs contribute to the development and progression of HF, and to assess their emerging utility as diagnostic biomarkers and therapeutic candidates. Sources were identified through searches in MEDLINE via Ovid (last accessed October 2025), Scopus (last accessed October 2025), Web of Science (last accessed October 2025), and Google Scholar (last accessed November 2025). The search strategy applied the terms “heart failure with preserved ejection fraction” or “HFpEF” or “diastolic heart failure” combined with “non-coding RNA” or “microRNA” or “miRNA” or “lncRNA” or “long non-coding RNA” or “circRNA” or “circular RNA” or “small nucleolar RNA”. Human studies were prioritized, and work involving rodent or other animal models was included when relevant mechanistic insight was provided. Search outputs from MEDLINE, Scopus, and Web of Science were exported in RIS or BibTeX format for screening.

Inclusion required that studies reported original data linking non-coding RNA expression or function to HFpEF, defined by preserved LVEF and diastolic impairment according to clinical guideline criteria, or by established phenotypes in experimental models. Articles needed to present extractable data illustrating regulatory roles, diagnostic or prognostic associations, or therapeutic modulation of microRNAs. Only peer-reviewed full-text manuscripts written in English were incorporated. There were no formal date restrictions, but most selected material was published between 2000 and early 2025. Exclusion was applied when studies investigated heart failure with reduced ejection fraction without presenting HFpEF-specific results, or when analysis of non-coding RNAs was incidental and lacked expression or functional evaluation. Reviews, commentaries, editorials, protocols, and conference abstracts without a complete dataset were omitted. Duplicate reports from shared cohorts were removed unless additional analysis justified inclusion. All records were screened for relevance by two reviewers trained in cardiovascular molecular biology, and discrepancies were resolved by discussion. Articles meeting the predetermined criteria were evaluated for methodology, sample characteristics, analytical approach, and reported outcomes relating to microRNA-mediated regulation in HFpEF progression, with emphasis on reproducibility and biological plausibility.

## 3. Pathophysiology and Molecular Mechanisms of Heart Failure with Preserved Ejection Fraction

HFpEF is primarily driven by abnormalities in diastolic relaxation, myocardial stiffness, and impaired energetic homeostasis rather than systolic pump failure. At the molecular level, its pathogenesis reflects a convergence of metabolic dysregulation, mitochondrial impairment, endothelial dysfunction, chronic low-grade inflammation, and interstitial fibrosis. Persistent neurohormonal activation promotes norepinephrine, angiotensin II, and aldosterone release, initiating vasoconstriction, hypertrophy, and redox imbalance. As oxidative burden increases, cardiomyocytes experience mitochondrial dysfunction, reduced fatty acid oxidation, and a shift toward glycolytic metabolism, leading to ATP deficiency and impaired diastolic relaxation [30,31]. Disrupted calcium cycling prolongs cytosolic calcium transients, delays myocardial relaxation, and activates hypertrophic transcriptional programs [30]. Excess reactive oxygen species amplify mitochondrial injury, impair electron transport chain function, inhibit ATP synthase activity, and induce ROS-driven ROS release. These events initiate apoptosis, stimulate fibroblast activation, and promote extracellular matrix accumulation, resulting in increased ventricular stiffness. Parallel inflammatory signaling, characterized by heightened TNF-α, IL-1, and IL-6, activates NFκB and matrix metalloproteinases, degrading extracellular structure and accelerating collagen deposition. Endothelial dysfunction further impairs nitric oxide bioavailability, exacerbates microvascular rarefaction, and worsens metabolic flexibility. Ultimately, these interconnected processes culminate in energetic failure, fibrosis, and maladaptive remodeling that define HFpEF.

**Figure 3:**
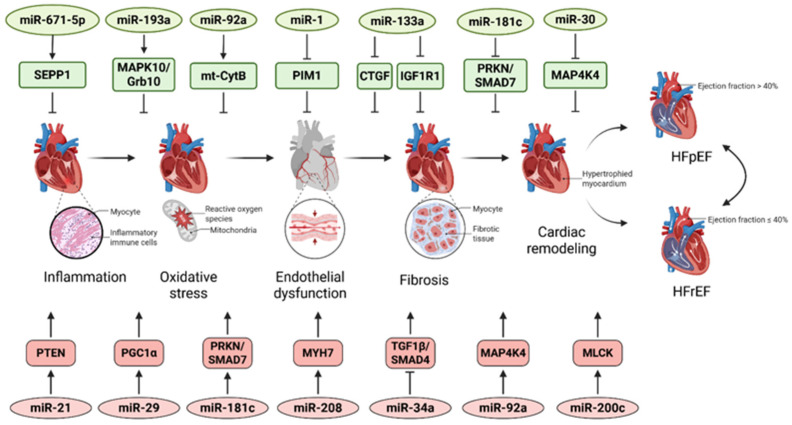
MicroRNA-mediated mechanisms in heart failure pathogenesis. The figure illustrates the principal miRNAs and their downstream targets that participate in the key processes driving heart failure, including inflammation, oxidative stress, endothelial dysfunction, fibrosis, and structural cardiac remodeling. MiRNAs and targets that promote these pathogenic pathways are shown in red, whereas those that exert protective or compensatory effects appear in green. Arrows denote potentiating or activating effects, while blunt arrows indicate inhibitory interactions. CTGF—Connective Tissue Growth Factor; Grb10—Growth Factor Receptor-Bound Protein 10; HFpEF—Heart Failure with Preserved Ejection Fraction; HFrEF—Heart Failure with Reduced Ejection Fraction; IGF1R1—Insulin-like Growth Factor 1 Receptor 1; MAP4K4—Mitogen-Activated Protein Kinase Kinase Kinase Kinase 4; MAPK10—Mitogen-Activated Protein Kinase 10; MLCK—Myosin Light Chain Kinase; mt-CytB—Mitochondrial Cytochrome B; PGC1α—Peroxisome Proliferator-Activated Receptor Gamma Coactivator 1 Alpha; PIM1—Proto-Oncogene, Serine/Threonine Kinase 1; PRKN—Parkin (E3 Ubiquitin-Protein Ligase); PTEN—Phosphatase and Tensin Homolog; SEPP1—Selenoprotein P; SMAD4—SMAD Family Member 4; SMAD7—SMAD Family Member 7; TGF1β—Transforming Growth Factor Beta 1.

MiRNAs are recognized as key epigenetic regulators that coordinate fibrotic, inflammatory, metabolic, and survival pathways in HFpEF. Distinct miRNA signatures sustain chronic inflammation and extracellular matrix expansion by modulating multiple targets simultaneously [32,33,34]. The involvement of selected miRNAs and their regulatory targets in HF pathogenesis is presented in Figure 3. In addition to miRNA-driven mechanisms, other epigenetic regulators contribute to metabolic and inflammatory control. Notably, recombinant Sirt1 supplementation has been shown to restore cardiac lipid homeostasis and prevent diabetes-related metabolic cardiomyopathy, underscoring the broader epigenetic landscape in HFpEF [35].

miR-21, enriched in cardiomyocytes and fibroblasts, drives TGF-β-dependent proliferation and fibrosis [32,36,37,38] and targets SPRY1 to enhance fibroblast survival [32,39,40] and extracellular matrix synthesis [32,41,42,43]. In patients with aortic stenosis, plasma and myocardial miR-21 concentrations correlated with histological fibrosis and impaired global longitudinal strain, demonstrating a direct mechanistic link between miR-21-mediated fibrogenesis and diastolic dysfunction [44].

Members of the miR-29 family suppress the expression of fibrillin, collagen, and elastin [32,41,45]. Their downregulation accelerates matrix accumulation by modulating TGF-β/SMAD signaling through TGFβ2 and MMP2, while SMAD3 acts as a transcriptional inhibitor of miR-29 [32,46].

miR-208a/b, transcribed from *MYH6*/*MYH7*, contributes to maladaptive remodeling during isoform switching toward MYH7 in hypertensive HF [32,47,48]. Elevated miR-208b promotes cardiomyocyte hypertrophy, AF onset, and fibrosis without stimulating proliferation [32,36], while stretch-activated miR-208a increases endoglin and collagen I production via TGF-β signaling [32,49].

Collectively, these miRNAs orchestrate the balance between injury and repair in cardiac tissue, influencing the degree of inflammation, fibrosis, and hypertrophy that shapes the clinical trajectory of HF.

## 4. Diagnostic Applications of microRNAs in Heart Failure

Circulating miRNAs are increasingly recognized as valuable biomarkers for detecting HF at both early and advanced stages due to their remarkable stability in the bloodstream and their close involvement in key molecular pathways, ensured by encapsulation in extracellular vesicles or binding to protective proteins, is particularly advantageous for clinical practice [20,50]. Their integration into multimarker diagnostic strategies enhances the performance of established biomarkers such as NT-proBNP, soluble suppressor of tumorigenicity-2 (sST2), galectin-3, and high-sensitivity troponins, improving recognition of myocardial stress, fibrosis, inflammation, oxidative stress, and cardiomyocyte injury [20,51,52]. When incorporated into liquid biopsy approaches, miRNAs offer molecular information beyond that provided by conventional tools and hold potential to improve sensitivity and specificity, particularly in heterogenous forms such as HFpEF where echocardiography and natriuretic peptides lack precision [20,50,51,52,53,54,55,56,57,58]. Numerous studies confirm that circulating miRNA profiles can effectively distinguish HF phenotypes (Table 1, Supplementary Table S1).

Combined miRNA panels consistently demonstrate strong diagnostic capacity, with AUC values often above 0.75 when differentiating HFpEF from healthy individuals and exceeding 0.80 when compared with HFrEF [54,95,96,97]. Their relevance is closely tied to their involvement in cardiac pathology, supporting their use for both detection and phenotype classification.

Among the best studied candidates, miR-21-5p displays distinct expression across HF types (Table 1). Levels are markedly lower in HFpEF than in HFrEF and correlate with diastolic dysfunction, myocardial hypertrophy, and NT-proBNP, showing early elevation before declining as disease progresses [54,63,64]. The let-7 family (let-7b-5p, let-7e-5p) also provides diagnostic information, the expression reflecting blood pressure, hypertrophy severity and remodeling, and displaying sex-related differences potentially linked to estrogen signaling [54,66,67].

A structured workflow has been introduced for miRNA biomarker development, including differential expression screening, phenotype correlation, discriminant model construction, and ROC validation [54,70,71,72]. Using this approach, a four-miRNA panel (let-7b-5p, let-7e-5p, miR-21-5p, and miR-140-3p) achieved AUC values above 0.9 in preclinical HFpEF and successfully differentiated HFpEF from healthy and HFrEF cohorts in clinical datasets [54,73] (Table 1). Meta-analytic data further support multi-marker strategies: for HFrEF, an eight-miRNA panel reached sensitivity of 85% and specificity of 88% (AUC 0.91), while a seven-miRNA signature for HFpEF achieved 82% sensitivity and 61% specificity (AUC 0.79) [20,74].

Among individual biomarkers, miR-423-5p remains the most consistently validated, correlating strongly with NT-proBNP and systolic function, and achieving AUC values around 0.86–0.91 in independent studies [20,59,60,61,75,98] (Table 1). Its stability despite comorbidities such as obesity and renal dysfunction strengthens its utility where natriuretic peptides may be misleading [60,61].

Several additional miRNAs hold diagnostic potential. A five-miRNA combination (miR-133a-3p, miR-378, miR-1-3p, miR-106b-5p, miR-133b) performed comparably to NT-proBNP and was linked to remodeling pathways such as MAPK, ErbB, and TGF-β [20,75]. In HFrEF, upregulation of multiple circulating miRNAs (miR 21-3p, miR 21-5p, miR 106b-5p, miR 23a-3p, miR 208a-3p, miR 1-3p, miR-126-5p, miR -133, and miR-223-3p) correlated with chamber dilation and hypertrophy [75], while experimental data indicate that miR-378 may suppress hypertrophy and fibrosis via MAPK/p38 inhibition [75,76]. More recently, miR-3135b, miR-3908, and miR-5571-5p have been identified as particularly promising markers, with discriminatory performance comparable or exceeding NT-proBNP and strong capacity to differentiate HFpEF [20,77] (Table 1).

Cardiomyocyte-derived miRNAs also serve as rapid diagnostic markers. MiR-1 and miR-133a are detectable within 1–3 h of ischemic onset, whereas miR-208b and miR-499 peak later. miR-499 achieves high diagnostic accuracy in distinguishing ACS from stable ischemia and differentiating STEMI from NSTEMI [20,89].

In summary, circulating miRNAs constitute a layered biomarker system capable of distinguishing HF phenotypes, reflecting disease mechanisms and supporting risk stratification. Broader clinical implementation will require standardized processing, reduced inter-laboratory variability and validation in large, diverse patient cohorts, particularly considering comorbidities, sex-specific differences and heterogeneity of HF presentation [20,99]. Integrative multi-miRNA panels are expected to enhance diagnostic precision and enable more personalized management approaches.

## 5. Prognostic Applications of microRNAs in Heart Failure

Circulating miRNAs provide clinically relevant information for predicting outcomes, stratifying risk, and monitoring disease progression. Variations in their expression correlate with mortality, rehospitalization, response to therapy, and degree of cardiac remodeling, making them suitable prognostic tools across the HF spectrum (Supplementary Table S1) [20]. Low admission levels of miR-423-5p are associated with markedly higher mortality and rehospitalization [22,98], while miR-30d independently predicts adverse outcomes and contributes to assessing remodeling reversal during cardiac resynchronization therapy via MAP3K4-mediated hypertrophy modulation [20,79] (Table 1).

Distinct miRNA signatures can guide therapeutic response prediction. Decreased myocardial miR-208a-3p, miR-208b-3p, miR-21-5p, and miR-199a-5p, together with increased miR-1-3p, associate with favorable remodeling during β-blocker treatment [20,100]. In a large cohort, miR-132 independently predicted rehospitalization and enhanced risk stratification beyond standard models [20,80]. A five-miRNA panel (miR-26b-5p, miR-145-5p, miR-92a-3p, miR-30e-5p, and miR-29a-3p) characterized responders to resynchronization therapy [20,81], with protective roles reported for miRNA-26b and miR-145 against hypertrophy and apoptosis, while miRNA-29a-3p and miRNA-30e-5p reduce collagen deposition [81,82,83,84,85,86,87,88] (Table 1).

Population studies reinforce prognostic value. Elevated miR-21 and miR-29a associate with increased all-cause, cardiovascular, cancer mortality, while low miR-126 predicts higher overall mortality [32,101]. In hypertensive cardiomyopathy, six miRNAs (miR-16, miR-20b, miR-93, miR-106b, miR-223, miR-423-5p) rose with HF progression and correlated with NT-proBNP and MYH7 expression [32,48] (Table 1).

MiRNAs involved in microvascular dysfunction contribute to diastolic impairment and HFpEF. miR-30 family members drive oxidative stress and impaired nitric oxide signaling [32,102]; miR-34a-5p and miR-92a-3p rise in chronic HFpEF, particularly in metabolic disease, promoting endothelial–mesenchymal transition and vascular remodeling [32,103]. Reduced endothelial miR-126a associates with diminished cardiac output and aggravated microvascular rarefaction [32,104]. In Takotsubo cardiomyopathy, co-expression of miR-16 and miR-26a heightens catecholamine sensitivity, linking emotional stress to acute cardiac dysfunction [32,90] (Table 1).

Altogether, circulating miRNAs offer multidimensional prognostic insight into HF, often outperforming conventional biomarkers by capturing early molecular disturbances. Their integration into clinical assessment may enhance risk stratification, guide treatment decisions, and support long-term monitoring in HF management [20].

## 6. Therapeutic Applications of microRNAs in Heart Failure

Circulating miRNAs are emerging as responsive biomarkers that reflect the molecular effects of HF therapies and help distinguish treatment responders (Supplementary Table S1) [20]. Although no miRNA-based drugs have been approved yet, antisense inhibitors- and mimetic approaches hold promise, highlighted by the development of the locked nucleic acid (LNA)-modified miR-132-3p inhibitor CDR132L, which has shown encouraging early clinical results [32].

Therapeutic modulation of miRNA expression is particularly evident with sodium/glucose cotransporter 2 (SGLT2) inhibitors. In HFpEF with diabetes, empagliflozin markedly reduced miR-21 and miR-92a levels, with normalization accompanying improved endothelial function, while other dysregulated miRNAs (miR-126, miR-342-3p, miR-638) remained unchanged under metformin or insulin therapy [20,91,93]. Preclinical studies show that empagliflozin and dapagliflozin induce distinct miRNA responses, with empagliflozin increasing miR-146a and miR-34a and dapagliflozin elevating only miR-146a, indicating differing cardioprotective pathways [93,94] (Table 1).

ARNI therapy (sacubitril/valsartan) produces a characteristic miRNA pattern associated with reverse remodeling. In HFpEF, ARNI induced miR-29b-3p, miR-221-3p, and miR-503-5p, particularly in patients with high baseline expression, with changes correlating with functional and fibrosis-related indices [20,94]. Bioinformatic and experimental data suggest that these miRNAs converge on PI3K/AKT signaling, and suppression of miR-29b-3p enhances cardiomyocyte survival and associates with decreased septal thickness and improved global strain after therapy [20,94]. Overlap between drug-modulated pathways (e.g., dapagliflozin effects on TGF-β/SMAD and inflammasome signaling) and miRNA-regulated networks such as miR-29 and miR-21 highlights potential synergy between pharmacologic and miRNA-targeted interventions [32] (Table 1).

Overall, circulating miRNAs act as sensitive indicators of therapeutic response, mirror remodeling processes and endothelial recovery, and provide mechanistic insight into cardioprotective drug action. While promising, miRNA-based interventions remain in early stages, and their clinical translation is limited by challenges in delivery, potential off-target effects, and regulatory hurdles. Nevertheless, current evidence, including the modulation of miRNAs by SGLT2 inhibitors and ARNI therapy, highlights their capacity to provide mechanistic insights, identify treatment responders, and guide personalized therapy. Integration of circulating miRNAs into clinical evaluation, alongside conventional biomarkers, may support personalized therapy selection and guide future miRNA-based treatment strategies.

## 7. Conclusions

The study of miRNAs in the development of HF is a promising frontier in cardiology, offering new opportunities for both diagnosis and therapy. MiRNAs exert a significant influence on the regulation of genes involved in cardiomyocyte function, cardiac remodeling, and inflammatory processes. A deeper understanding of their mechanisms of action may facilitate the development of effective biomarkers and targeted therapeutic strategies capable of slowing HF progression and improving patients’ quality of life. Integrating miRNA profiling into clinical practice holds the potential to advance truly personalized therapeutic approaches, enabling drug selection and dosing to be tailored according to the molecular response of individual patients.

Nevertheless, current evidence is limited by substantial heterogeneity across HF phenotypes, differences in study populations, and variability in analytical platforms, all of which complicate comparisons and hinder clinical translation. The field continues to face major obstacles, including the need for rigorous standardization of miRNA extraction and quantification, persistent inter-laboratory variability, and the limited scope and methodological inconsistency of existing clinical trials. Many published studies remain exploratory, with inconsistent replication and limited longitudinal data, leaving important gaps in clinical validation. Future research should prioritize standardized measurement protocols, large prospective cohorts representing diverse HF phenotypes, and integrative analyses linking miRNA signatures with imaging, hemodynamic, and genetic data to clarify their prognostic and mechanistic relevance.

Despite their high specificity, circulating miRNAs should be considered complementary rather than alternative biomarkers. Their clinical implementation requires standardized detection methods, validation in large multicenter studies, and demonstration of added value when combined with established biomarkers, as part of a precision medicine approach to managing patients with HF [87].

## Figures and Tables

**Table 1:** MicroRNAs with major diagnostic, prognostic and therapeutic relevance in heart failure.

miRNA/Panel	Primary Role	HF Phenotype	Diagnostic or Prognostic Value	Clinical Implications	References
miR-423-5p	Diagnostic, prognostic	HFrEF, HFpEF	AUC 0.86–0.91; sensitivity 0.66–0.81; specificity 0.67–0.84; correlates with NT-proBNP and EF	Stable across comorbidities; low levels predict mortality and rehospitalization	[20,59,60,61,62]
miR-21-5p	Diagnostic, prognostic, therapeutic response	HFpEF, fibrosis-related HF	AUC 0.94; sensitivity 80; specificity 0.91	Dynamic over disease course; modulated by SGLT2 inhibitors; predicts hospitalization and mortality	[20,32,54,63,64,65]
let-7b-5p/let-7e-5p	Diagnostic	HFpEF (hypertension-associated)	AUC > 0.9, correlates with BP, hypertrophy, remodeling	Sex-related differences reported; component of validated HFpEF panel	[54,66,67,68]
miR-140-3p	Diagnostic	HFpEF	Included in 4-miRNA HFpEF panel with AUC > 0.9	Strong discrimination HFpEF vs. HFrEF and controls	[69]
4-miRNA HFpEF panel: let-7b-5p, let-7e-5p, miR-21-5p, miR-140-3p	Diagnostic	Chronic HFpEF	AUC > 0.9; validated cross-species	Effective for rule-in and rule-out strategies	[54,70,71,72,73]
8-miRNA HFrEF panel: miR-18b-3p, miR-21-5p, miR-22-3p, miR-92b-3p, miR-129-5p, miR-320a-5p, miR-423-5p, miR-675-5p	Diagnostic	Chronic HFrEF	Sensitivity 0.85; specificity 0.88; AUC 0.91	Meta-analysis confirmed efficacy	[20,74]
7-miRNA HFpEF panel: miR-19b-3p, miR-30c-5p, miR-206, miR-221-3p, miR-328-5p, miR-375-3p, miR-424-5p	Diagnostic	Chronic HFpEF	Sensitivity 0.82, specificity 0.61, AUC 0.79	Potential for wide diagnostic implementation	[20,74]
miR-133a-3p, miR-106b-5p, miR-1-3p, miR-133b, miR-378 (5-miRNA panel)	Diagnostic	High-risk HF/HFrEF	AUC 0.99	Targets MAPK/ErbB/TGF-β pathways; correlates with LV function	[20,75,76]
miR-133a-5p, miR-1-3p, miR-106b-5p, miR-126-5p, miR-195-5p	Diagnostic	Myocardial hypertrophy, remodeling	Correlates with LVEF and structural indices	Indicates structural deterioration	[75]
miR-3135b, miR-3908, miR-5571-5p	Diagnostic	HFpEF vs. HFrEF differentiation	miR-3135b AUC 1.00; miR-5571-5p AUC 0.94	Could complement NT-proBNP	[20,77]
miR-30d	Prognostic	HF outcomes prediction	Independent mortality predictor (AUC 0.81)	Indicates reverse remodeling under CRT	[78,79]
miR-132	Prognostic	Rehospitalization risk	HR 0.79; improves risk models (cNRI 0.205)	Robust large-cohort validation	[20,80]
CRT-responder panel: miR-26b-5p, miR-145-5p, miR-92a-3p, miR-30e-5p, miR-29a-3p	Prognostic/therapy-response	Response to CRT	Differential expression predicts responders	Anti-fibrotic and anti-hypertrophic effects	[20,81,82,83,84,85,86,87,88]
Cardiomyocyte-specific miRNAs: miR-1, miR-133a, miR-208b, miR-499	Early injury markers	ACS/acute HF	Peak within hours; miR-499 AUC 0.93 for ACS	Enable rapid triage and injury detection	[20,89]
miR-30 family, miR-34a-5p, miR-92a-3p, miR-126	Prognostic	HFpEF, microvascular injury	Linked to NO signaling, oxidative stress	Reflect endothelial dysfunction	[81]
miR-16, miR-26a	Prognostic	Takotsubo cardiomyopathy	Stress-responsive co-expression pattern	Connects catecholamine stress to dysfunction	[32,90]
miR-21, miR-92a (SGLT2-modulated)	Therapeutic	HFpEF + diabetes	Normalize after empagliflozin	Changes correlate with vascular improvement	[20,91,92,93,94]
miR-146a, miR-34a (SGLT2-responsive)	Therapeutic	T2DM-associated HF	Differential modulation by empagliflozin vs. dapagliflozin	Drug-specific signaling signatures	[20,91,92,93,94]
miR-29b-3p, miR-221-3p, miR-503-5p (ARNI-responsive)	Therapeutic	HFpEF under sacubitril/valsartan	Downregulated after 6 months therapy	miR-29b-3p reduction improves fibrosis metrics	[20,94]
CDR132L (miR-132-3p inhibitor)	Therapeutic agent	Experimental HF therapy	First human trials promising	miRNA-targeted treatment candidate	[32]

ACS—acute coronary syndrome; ARNI—angiotensin receptor–neprilysin inhibitor; AUC—area under the ROC curve; CRT—cardiac resynchronization therapy; HF—heart failure; HFpEF—heart failure with preserved ejection fraction; HFrEF—heart failure with reduced ejection fraction; LV—left ventricle; LVEF—left ventricular ejection fraction; MAPK/ErbB/TGF-β—mitogen-activated protein kinase/erythroblastic leukemia viral oncogene/TGF-β signaling pathways; miRNA—microRNA; NO—nitric oxide; NT-proBNP—N-terminal pro-B-type natriuretic peptide; SGLT2—sodium–glucose cotransporter-2.

## Data Availability

No new data were created or analyzed in this study. Data sharing is not applicable to this article.

## Supplementary Materials

The following supporting information can be downloaded at: https://www.mdpi.com/article/10.3390/ijms262412085/s1. Refs. [105,106,107,108,109,110,111,112,113,114,115,116,117,118,119,120,121,122,123,124,125,126,127,128,129,130,131,132,133,134] are cited in the Supplementary Materials file.

## Abbreviations

The following abbreviations are used in this manuscript:

ACS	Acute coronary syndrome
AF	Atrial fibrillation
AUC	Area under the curve
CAD	Coronary artery disease
GWAS	Genome-wide association study
HF	Heart failure
HFimpEF	Heart failure with improved ejection fraction
HFmrEF	Heart failure with mildly reduced ejection fraction
HFpEF	Heart failure with preserved ejection fraction
HFrEF	Heart failure with reduced ejection fraction
lncRNA	Long non-coding RNA
LVEF	Left ventricular ejection fraction
MI	Myocardial infarction
miRNA	MicroRNA
NT-proBNP	N-terminal pro-B-type natriuretic peptide
PRS	Polygenic risk score
