# Supplementary material for: The Role of MicroRNAs in the Pathophysiology and Management of Heart Failure: From Molecular Mechanisms to Clinical Application
Source: Int J Mol Sci. 2025 Dec 16;26(24):12085. doi: 10.3390/ijms262412085 (PMC12733101; doi:10.3390/ijms262412085)
Supplement: Supplementary file 1 [file ijms-26-12085-s001.zip › ijms-4012547-supplementary.pdf]

Supplementary Table S1. Circulating microRNAs with diagnostic, prognostic and therapeutic potential.

miRNA	Regulation	Action	Value	AUC	Sensitivity	Specificity	Phenotype	Ref.
MyomiRs (muscle-enriched regulators of myogenesis and cardiac function)								
miR-1	Increased	Indicator of myocardial damage	Diagnostic	0.86	0.88	0.802	ACS, HF	[1,2]
	Increased	Associated with more severe cardiac damage during ischemia	Prognostic	0.62	0.80	0.503	ACS, HF	[1,2]
	Increased	Overexpression may reverse myocardial hypertrophy	Therapeutic	-	-	-	HF	[3]
miR-133	Increased	Indicator of myocardial damage	Diagnostic	0.86	0.82	0.81	ACS, HF	[1,3,4]
	Decreased	Promoted therapeutic myocardial remodeling by inhibiting fibrosis, inflammation, and compensatory hypertrophy in ischemic hearts	Therapeutic	-	-	-	HF	[3]
miR-206	Decreased	Elevated in myocardial tissue during infarction and HFrEF	Diagnostic	0.72	0.69	0.658	HFpEF	[5]
miR-208	Increased	Indicator of myocardial damage	Diagnostic	0.93	0.83	0.97	ACS, HF	[1,6]
miR-208b	Increased	Indicator of myocardial damage	Diagnostic	0.88	0.78	0.95	ACS, HF	[1,6]
	Increased	Predicts adverse outcomes, including mortality and HF progression after acute MI6	Prognostic	0.93	-	-	ACS, HF	[1]
miR-499	Increased	Early marker of cardiomyocyte injury	Diagnostic	0.86	0.80	0.8028	Acute HF, ACS[2]	[1,7,8]
Metabolic and energy-homeostasis associated miRNAs (including lipid metabolism, insulin signaling)								
miR-103	Increased	Induced by hypoxia; involved in pyruvate and lipid metabolism; associated with HF[1]	Diagnostic	0.64	0.90	0.750	Acute HF	[7,9]
miR-122-5p	Increased	Associated with elevated mortality risk	Prognostic	0.67	-	-	HFrEF	[7,10]
miR-22	Increased	MiRNA-coated stents reduced inflammation and extracellular matrix deposition, significantly inhibiting in-stent restenosis	Therapeutic	-	-	-	ACS, HF, restenosis	[1]
miR-22-3p	Increased	Distinguishes patients with and without post-infarction left ventricular remodeling	Diagnostic	0.90	0.90	0.9	HFrEF	[5]
miR-223	Increased	Associated with increased risk of mortality, MACE5, and HF progression; effects may vary between pro- and anti-hypertrophic actions	Prognostic	0.73	0.88	0.57	ACS, HF	[1,11]
miR-223-3p	Increased	Indicator of myocardial damage	Therapeutic	-	0.70	82.61	ACS, HF	[1,12]
miR-320a-3p	Increased	Overexpression in cardiac fibroblasts inhibits fibrosis and hypertrophy	Therapeutic	0.87	0.76	0.895	HF	[3,13]
miR-320a-5p	Increased	Differentiates HF subtypes; predicts mortality risk	Diagnostic	-	-	-	HFrEF	[5]

miR-375-3p	Decreased	Differentiates HFrEF from HFpEF; also useful in acute myocardial infarction	Diagnostic	-	-	-	HFpEF	[5]
Hypertrophic drivers (promote cardiomyocyte growth and pathological remodeling through MAPK, PI3K–Akt, calcineurin–NFAT and fetal gene programming)								
miR-21	Increased	Correlates with disease severity, rehospitalization, and cardiovascular mortality<br>Induced macrophage polarization from pro-inflammatory to reparative, stimulating angiogenesis and limiting fibrosis and myocardial remodeling	Prognostic<br>Therapeutic	0.75	0.56	0.85	ACS, HF	[1,14]
miR-21-5p	Increased	Marker of cardiac inflammation	Diagnostic	0.94	0.80	0.91	HFrEF	[5]
miR-17-3p	Increased	Associated with diabetic cardiomyopathy	Diagnostic	0.86	0.85	0.86	HF	[3,15]
miR-18a-5p	Decreased	Associated with reduced overall survival in HF patients	Prognostic	0.89	0.87	0.79	HF	[16,17]
miR-18b-3p	Increased	Diagnostic biomarker for HF, especially HFrEF	Diagnostic	0.86	0.87	0.85	HFrEF,	[5]
miR-19a-3p	Increased	Independent biomarker for HFpEF[3]	Diagnostic	0.99	-	-	HFpEF	[7,18]
miR-19b	Decreased	Correlates with NT-proBNP and adverse clinical outcomes;	Diagnostic	0.85	0.77	0.79	HFrEF[4]	[7,19]
miR-19b-3p	Decreased	Strongly associated with left ventricular hypertrophy and fibrosis; implicated in disease pathogenesis	Diagnostic	-	0.86	0.61	HFpEF	[5]
miR-27a-3p	Increased	Linked to poor outcomes in both acute and chronic HF; associated with increased mortality risk	Prognostic	0.69	-	-	HFrEF	[7,10]
miR-92b-3p	Increased	Dysregulated in cardiac hypertrophy and other cardiac conditions	Diagnostic	-	-	-	HFrEF	[5]
miR-30	Decreased	Associated with adverse outcomes; helps differentiate HF subtypes<br>Predicts risk of in-stent restenosis in coronary arteries	Prognostic<br>Therapeutic	0.63	-	-	ACS, HF	[1,7,9,16]
miR-30b-5p	Decreased	Predicts risk of in-stent restenosis in coronary arteries<br>Associated with reduced overall survival in HF patients	Therapeutic	0.80	-	-	ACS, HF	[1,16]
miR-30c-5p	Decreased	Differentiates HFrEF from HFpEF	Diagnostic	0.80	0.83	0.61	HFpEF	[5]
Anti-remodeling and pro-survival regulators (restrain hypertrophy, reduce apoptosis and support mitochondrial/FOXO-linked adaptive responses)								
miR-145	Decreased	Marker of myocardial injury; correlates positively with BNP and negatively with ejection fraction;<br>Inflammation-associated linked to poorer outcomes	Diagnostic<br>Prognostic	0.72	0.60	0.78	ACS, HF	[1,20]
miR-145-5p	Increased	Linked to all-cause mortality in HF patients	Prognostic	0.92	0.82	0.94	HF	[16,21]
miR-124	Increased	Promotes systemic inflammation; M2 macrophage polarization; influences reverse cardiac remodeling	Diagnostic	0.79	0.60	0.95	ACS, HF	[1,22]

miR-127	Increased	Promotes systemic inflammation; M1 macrophage polarization; affects cardiac remodeling	Diagnostic	0.69	0.60	0.78	HFpEF	[3]
miR-132	Decreased	Led to reverse myocardial remodeling, decreased fibrosis, and mitigated maladaptive hypertrophy	Therapeutic	0.74	-	-	HF	[3,23]
miR-212	Decreased	Reduced myocardial hypertrophy and slowed HF progression	Therapeutic	0.75	-	-	HF	[3]
miR-342	Decreased	Predicts poorer outcomes, including mortality; involved in cardiac repair and functional recovery after MI[8]<br>Inflammation-associated; strong univariate predictor of mortality	Prognostic				Acute HF, ACS, HF	[1,7]
miR-129-5p	Increased	Dysregulated in HF patients<br>Associated with HF and reduced survival	Diagnostic<br>Prognostic	0.92	0.96	0.77	HFrEF	[5]
miR-9-5p	Increased	Promotes systemic inflammation; M1 macrophage polarization; affects cardiac remodeling	Diagnostic	0.91	0.81	0.87	HFpEF	[3,24]
Fibrosis/ extracellular matrix modulators (control fibroblast activation, collagen deposition and TGF-β/SMAD signaling; influence extracellular matrix turnover)								
miR-125b	Increased	Promotes systemic inflammation; macrophage polarization; influences cardiac remodeling	Diagnostic	0.85	0.81	0.74	HFpEF	[3,25]
miR-146a	Increased	Promotes systemic inflammation; M2 macrophage polarization; influences cardiac remodeling<br>Inflammation-associated; predicts adverse outcomes in HF and related conditions	Diagnostic<br>Prognostic	0.85	0.89	0.81	HFpEF	[1,3,26]
miR-150	Decreased	Predicts adverse LV remodeling after acute MI	Prognostic	-	-	-	ACS, HF	[1]
miR-150-5p	Increased	Related to overt heart failure occurrence	Diagnostic	0.97	0.96	0.96	ATTRm[7]	[7,27]
miR-34a	Increased	Promotes cardiomyocyte apoptosis; M2 macrophage polarization<br>Correlates with worse prognosis in HF; linked to LV remodeling, cardiac fibrosis, and cardiomyocyte death	Diagnostic<br>Prognostic	.90	0.76	0.90	HF, HFpEF	[1,3,28]
miR-590-3p	Increased	Associated with risk factors worsening outcomes, such as atrial fibrillation and left atrial enlargement	Prognostic	0.62	-	-	HFrEF	[7,10]
miR-675-5p	Increased	Detects HFrEF with high sensitivity and specificity	Diagnostic	0.89	0.77	0.87	HFrEF	[5]
Angiogenic/vascular regulators (maintain endothelial function and neovascularisation under ischemic stress via VEGF, eNOS and Notch-related pathways)								
miR-126	Decreased	Correlates with endothelial dysfunction	Diagnostic	0.81	-	-	Ischemic HF	[7,29]
miR-221	Decreased	Associated with impaired cardiac function; negatively impacts angiogenesis; linked to fibrosis and hypertrophy;	Diagnostic<br>Prognostic	0.76	0.71	0.67-	HFpEF, ACS, HF	[1,5,30]

		High levels predict worse prognosis, particularly sudden cardiac death and HF progression; low levels linked to increased fibrosis and stiffness in advanced HF						
miR-424-5p	Increased	Diagnoses chronic HF; differentiates HF subtypes; adds value when combined with NT-proBNP	Diagnostic	0.71	0.85	0.59	HFpEF	[5]
Inflammatory/immune regulators (control cytokine production, immune-cell recruitment and inflammatory remodeling via NF-κB and JAK–STAT)								
miR-142-3p	Decreased	Linked to poor outcomes in acute HF; may have a protective role in myocardial injury, with prognostic effects varying by cardiac condition	Prognostic	0.73	0.78	0.68	Acute HF	[7,26]
miR-155	Increased	Promotes systemic inflammation; M1 macrophage polarization; affects cardiac remodeling	Diagnostic	-	0.85	0.65	HFpEF	[3]
miR-181b	Increased	Marker of cardiomyocyte hypertrophy	Diagnostic	0.97	-	-	HF	[3,31]
miR-190a	Decreased	Associated with adverse clinical outcomes	Prognostic	0.86	0.84	0.80-	Chronic HF	[7,32]
Stress/hypoxia-response regulators (mediate HIF-1α-dependent survival, oxidative stress responses and autophagy under ischemic load)								
miR-210	Increased	Correlates with increased mortality, especially in acute HF	Prognostic	0.63	-	-	Chronic HF	[7,10]
miR-24	Increased	Associated with smaller necrotic areas and improved cardiac function	Therapeutic	-	-	-	HF	[3]
miR-423-5p	Increased	Released during myocardial apoptosis in HF	Diagnostic	0.86	0.81	0.67	Acute HF	[7,33,34]
	Decreased	Associated with reduced overall survival in HF patients	Prognostic	0.78	0.66	0.84	HF	[16,35]
miR-328-5p	Decreased	Diagnostic and predictive biomarker; involved in electrical remodeling; correlates with arrhythmia severity	Diagnostic	-	-	-	HFpEF	[5]
miR-339-3p	Increased	Potential biomarker for senile cardiac amyloidosis	Diagnostic	0.67	0.36	0.88	ATTRwt[6]	[7,36]
Combined								
Panel: miR-1, miR-21, miR-23 and miR-423-5-p	Increased	Indicator of myocardial damage	Diagnostic	0.84	0.77	0.98	ACS, HF	[1,37]
Panel: miR-18b-3p, miR-21-5p, miR-22-3p, miR-92b-3p, miR-129-5p, miR-320a-5p, miR-423-5p, miR-675-5p	Increased	Diagnostic biomarker for HF	Diagnostic	0.91	0.85	0.88	HFrEF, HFpEF	[5]

Panel: miR-19b-3p, miR-30c-5p, miR-206, miR-221-3p, miR-328-5p, miR-375-3p, miR-424-5p	Increased	Diagnostic biomarker for HF	Diagnostic	-	0.82	0.61	HFrEF	[5]

ACS – acute coronary syndrome; ATTRwt – wild-type transthyretine amyloidosis; ATTRm – hereditary transthyretine amyloidosis; HF – heart failure; HFpEF – heart failure with preserved ejection fraction; HFrEF – heart failure with reduced ejection fraction; LV – left ventricle; MACE5 – five-point major adverse cardiovascular event; MI –myocardial infarction; NT-proBNP – N-terminal pro-B-type natriuretic peptide.

References:

1. Kabłak-Ziembicka, A.; Badacz, R.; Okarski, M.; Wawak, M.; Przewłocki, T.; Podolec, J. Cardiac microRNAs: diagnostic and therapeutic potential. *Archives of medical science : AMS* **2023**, *19*, 1360-1381, doi:10.5114/aoms/169775.
2. Su, T.; Shao, X.; Zhang, X.; Han, Z.; Yang, C.; Li, X. Circulating microRNA-1 in the diagnosis and predicting prognosis of patients with chest pain: a prospective cohort study. *BMC Cardiovasc Disord* **2019**, *19*, 5, doi:10.1186/s12872-018-0987-x.
3. D'Amato, A.; Prosperi, S.; Severino, P.; Myftari, V.; Correale, M.; Perrone Filardi, P.; Badagliacca, R.; Fedele, F.; Vizza, C.D.; Palazzuoli, A. MicroRNA and Heart Failure: A Novel Promising Diagnostic and Therapeutic Tool. *J Clin Med* **2024**, *13*, doi:10.3390/jcm13247560.
4. Guo, M.; Luo, J.; Zhao, J.; Shang, D.; Lv, Q.; Zang, P. Combined Use of Circulating miR-133a and NT-proBNP Improves Heart Failure Diagnostic Accuracy in Elderly Patients. *Medical science monitor : international medical journal of experimental and clinical research* **2018**, *24*, 8840-8848, doi:10.12659/msm.911632.
5. Parvan, R.; Hosseinpour, M.; Moradi, Y.; Devaux, Y.; Cataliotti, A.; da Silva, G.J.J. Diagnostic performance of microRNAs in the detection of heart failure with reduced or preserved ejection fraction: a systematic review and meta-analysis. *European journal of heart failure* **2022**, *24*, 2212-2225, doi:10.1002/ejhf.2700.
6. Wang, J.; Xu, L.; Tian, L.; Sun, Q. Circulating microRNA-208 family as early diagnostic biomarkers for acute myocardial infarction: A meta-analysis. *Medicine* **2021**, *100*, e27779, doi:10.1097/md.0000000000027779.
7. Fumarulo, I.; De Prisco, A.; Salerno, E.N.M.; Ravenna, S.E.; Vaccarella, M.; Garramone, B.; Burzotta, F.; Aspromonte, N. New Frontiers of microRNA in Heart Failure: From Clinical Risk to Therapeutic Applications. *J Clin Med* **2025**, *14*, doi:10.3390/jcm14186361.
8. Zhang, L.; Chen, X.; Su, T.; Li, H.; Huang, Q.; Wu, D.; Yang, C.; Han, Z. Circulating miR-499 are novel and sensitive biomarker of acute myocardial infarction. *Journal of thoracic disease* **2015**, *7*, 303-308, doi:10.3978/j.issn.2072-1439.2015.02.05.
9. Ellis, K.L.; Cameron, V.A.; Troughton, R.W. Circulating microRNAs as candidate markers to distinguish heart failure in breathless patients. *European journal of …* **2013**, doi:10.1093/eurjhf/hft078.
10. Parvan, R.; Becker, V.; Hosseinpour, M.; Moradi, Y.; Louch, W.E.; Cataliotti, A.; Devaux, Y.; Frisk, M.; Silva, G.J.J. Prognostic and predictive microRNA panels for heart failure patients with reduced or preserved ejection fraction: a meta-analysis of Kaplan-Meier-based individual patient data. *BMC Med* **2025**, *23*, 409, doi:10.1186/s12916-025-04238-0.
11. Gager, G.M.; Eyileten, C.; Postuła, M.; Nowak, A.; Gąsecka, A.; Jilma, B.; Siller-Matula, J.M. Expression Patterns of MiR-125a and MiR-223 and Their Association with Diabetes Mellitus and Survival in Patients with Non-ST-Segment Elevation Acute Coronary Syndrome. *Biomedicines* **2023**, *11*, doi:10.3390/biomedicines11041118.

12. Kuai, Z.; Ma, Y.; Gao, W.; Zhang, X.; Wang, X.; Ye, Y.; Zhang, X.; Yuan, J. Potential diagnostic value of circulating miRNAs in HFrEF and bioinformatics analysis. *Heliyon* **2024**, *10*, e37929, doi:10.1016/j.heliyon.2024.e37929.
13. Han, Q.; Zhang, L.; Liao, R. Diagnostic and prognostic significance of miR-320a-3p in patients with chronic heart failure. *BMC Cardiovasc Disord* **2024**, *24*, 308, doi:10.1186/s12872-024-03966-0.
14. Wang, Y.; Liang, Y.; Zhao, W.; Fu, G.; Li, Q.; Min, X.; Guo, Y. Circulating miRNA-21 as a diagnostic biomarker in elderly patients with type 2 cardiorenal syndrome. *Sci Rep* **2020**, *10*, 4894, doi:10.1038/s41598-020-61836-z.
15. Xue, S.; Liu, D.; Zhu, W.; Su, Z.; Zhang, L.; Zhou, C.; Li, P. Circulating MiR-17-5p, MiR-126-5p and MiR-145-3p Are Novel Biomarkers for Diagnosis of Acute Myocardial Infarction. *Frontiers in physiology* **2019**, *10*, 123, doi:10.3389/fphys.2019.00123.
16. Yang, J.; Yang, X.S.; Fan, S.W.; Zhao, X.Y.; Li, C.; Zhao, Z.Y.; Pei, H.J.; Qiu, L.; Zhuang, X.; Yang, C.H. Prognostic value of microRNAs in heart failure: A meta-analysis. *Medicine* **2021**, *100*, e27744, doi:10.1097/md.0000000000027744.
17. Teng, P.; Liu, Y.; Zhang, M.; Ji, W. Diagnostic and Prognostic Significance of serum miR-18a-5p in Patients with Atherosclerosis. *Clinical and applied thrombosis/hemostasis : official journal of the International Academy of Clinical and Applied Thrombosis/Hemostasis* **2021**, *27*, 10760296211050642, doi:10.1177/10760296211050642.
18. Zhong, J.; He, Y.; Chen, W.; Shui, X.; Chen, C.; Lei, W. Circulating microRNA-19a as a potential novel biomarker for diagnosis of acute myocardial infarction. *Int J Mol Sci* **2014**, *15*, 20355-20364, doi:10.3390/ijms151120355.
19. Zhang, L.; Xu, R.L.; Liu, S.X.; Dong, S.H. Diagnostic value of circulating microRNA‐19b in heart failure. *European Journal of* … **2020**, doi:10.1111/eci.13308.
20. Faccini, J.; Ruidavets, J.B.; Cordelier, P.; Martins, F.; Maoret, J.J.; Bongard, V.; Ferrières, J.; Roncalli, J.; Elbaz, M.; Vindis, C. Circulating miR-155, miR-145 and let-7c as diagnostic biomarkers of the coronary artery disease. *Sci Rep* **2017**, *7*, 42916, doi:10.1038/srep42916.
21. Zhu, Q.; Li, S.; Ji, K.; Zhou, H.; Luo, C.; Sui, Y. Differentially expressed TUG1 and miR-145-5p indicate different severity of chronic heart failure and predict 2-year survival prognosis. *Experimental and therapeutic medicine* **2021**, *22*, 1362, doi:10.3892/etm.2021.10796.
22. Dimitry, M.O.; Soliman, Y.M.A.; ElKorashy, R.I.; Raslan, H.M.; Kamel, S.A.; Hassan, E.M.; Ahmed, F.E.; Yousef, R.N.; Awadallah, E.A. Role of micro-RNAs 21, 124 and other novel biomarkers in distinguishing between group 1 WHO pulmonary hypertension and group 2, 3 WHO pulmonary hypertension. *The Egyptian heart journal : (EHJ) : official bulletin of the Egyptian Society of Cardiology* **2023**, *75*, 76, doi:10.1186/s43044-023-00395-w.
23. Song, R.; Zhang, L. MicroRNAs and therapeutic potentials in acute and chronic cardiac disease. *Drug Discov Today* **2024**, *29*, 104179, doi:10.1016/j.drudis.2024.104179.
24. Liu, H.; Zhou, J.; Jiang, W.; Wang, F. Analysis of the diagnostic and prognostic value of miR-9-5p in carotid artery stenosis. *Bosnian journal of basic medical sciences* **2021**, *21*, 724-729, doi:10.17305/bjbms.2021.5545.
25. Xue, Q.; Yang, L.; Wang, J.; Li, L.; Wang, H.; He, Y. lncRNA ROR and miR-125b Predict the Prognosis in Heart Failure Combined Acute Renal Failure. *Dis Markers* **2022**, *2022*, 6853939, doi:10.1155/2022/6853939.
26. Scărlătescu, A.I.; Barbălată, T.; Sima, A.V.; Stancu, C.; Niculescu, L.; Micheu, M.M. miR-146a-5p, miR-223-3p and miR-142-3p as Potential Predictors of Major Adverse Cardiac Events in Young Patients with Acute ST Elevation Myocardial Infarction-Added Value over Left Ventricular Myocardial Work Indices. *Diagnostics (Basel, Switzerland)* **2022**, *12*, doi:10.3390/diagnostics12081946.
27. Vita, G.L.; Aguennouz, M.; Polito, F.; Oteri, R.; Russo, M.; Gentile, L.; Barbagallo, C.; Ragusa, M.; Rodolico, C.; Di Giorgio, R.M.; et al. Circulating microRNAs Profile in Patients With Transthyretin Variant Amyloidosis. *Front Mol Neurosci* **2020**, *13*, 102, doi:10.3389/fnmol.2020.00102.

28. Li, H.; Chen, M.; Feng, Q.; Zhu, L.; Bai, Z.; Wang, B.; Guo, Z.; Hou, A.; Li, H. MicroRNA-34a in coronary heart disease: Correlation with disease risk, blood lipid, stenosis degree, inflammatory cytokines, and cell adhesion molecules. *Journal of clinical laboratory analysis* **2022**, *36*, e24138, doi:10.1002/jcla.24138.
29. Ali, W.; Mishra, S.; Rizvi, A.; Pradhan, A.; Perrone, M.A. Circulating microRNA-126 as an Independent Risk Predictor of Coronary Artery Disease: A Case-Control Study. *Ejifcc* **2021**, *32*, 347-362.
30. Huang, D.; Chen, Z.; Wang, J.; Chen, Y.; Liu, D.; Lin, K. MicroRNA-221 is a potential biomarker of myocardial hypertrophy and fibrosis in hypertrophic obstructive cardiomyopathy. *Bioscience reports* **2020**, *40*, doi:10.1042/bsr20191234.
31. Huang, Y.M.; Li, W.W.; Wu, J.; Han, M.; Li, B.H. The diagnostic value of circulating microRNAs in heart failure. *Experimental and therapeutic medicine* **2019**, *17*, 1985-2003, doi:10.3892/etm.2019.7177.
32. Sun, B.; Meng, M.; Wei, J.; Wang, S. Long noncoding RNA PVT1 contributes to vascular endothelial cell proliferation via inhibition of miR-190a-5p in diagnostic biomarker evaluation of chronic heart failure. *Experimental and therapeutic medicine* **2020**, *19*, 3348-3354, doi:10.3892/etm.2020.8599.
33. Tijsen, A.J.; Creemers, E.E.; Moerland, P.D.; de Windt, L.J.; van der Wal, A.C.; Kok, W.E.; Pinto, Y.M. MiR423-5p as a circulating biomarker for heart failure. *Circ Res* **2010**, *106*, 1035-1039, doi:10.1161/circresaha.110.218297.
34. Yan, H.; Ma, F.; Zhang, Y.; Wang, C.; Qiu, D.; Zhou, K.; Hua, Y.; Li, Y. miRNAs as biomarkers for diagnosis of heart failure: A systematic review and meta-analysis. *Medicine* **2017**, *96*, e6825, doi:10.1097/md.0000000000006825.
35. Guo, X.; Zhou, Y.; Huang, H.; Zong, Z.; Xin, M.; Yang, K. Diagnostic and prognostic value of microRNA423-5p in patients with heart failure. *Journal of cardiothoracic surgery* **2024**, *19*, 550, doi:10.1186/s13019-024-03091-1.
36. Shevchenko, O.; Velikiy, D.; Sharapchenko, S.; Gichkun, O.E.; Marchenko, A.V.; Ulybysheva, A.A.; Pavlov, V.S.; Mozheiko, N.P.; Koloskova, N.N.; Shevchenko, A.O. Diagnostic value of microRNA-27 and -339 in heart transplant recipients with myocardial fibrosis. *Russian Journal of Transplantology and Artificial Organs* **2021**, *23*, 73-81, doi:10.15825/1995-1191-2021-3-73-81.
37. Sadat-Ebrahimi, S.R.; Rezabakhsh, A.; Aslanabadi, N.; Asadi, M.; Zafari, V.; Shanebandi, D.; Zarredar, H.; Enamzadeh, E.; Taghizadeh, H.; Badalzadeh, R. Novel diagnostic potential of miR-1 in patients with acute heart failure. *PloS one* **2022**, *17*, e0275019, doi:10.1371/journal.pone.0275019.
